# Supplementary material for: Effects of GABAAR modulators CL218872 and MRK-016 on neural repair and synaptic plasticity in mice with Intracerebral hemorrhage
Source: PLoS One. 2026 Mar 23;21(3):e0345025. doi: 10.1371/journal.pone.0345025 (PMC13008086; doi:10.1371/journal.pone.0345025)
Supplement: S1 — (PDF) [file pone.0345025.s001.pdf]

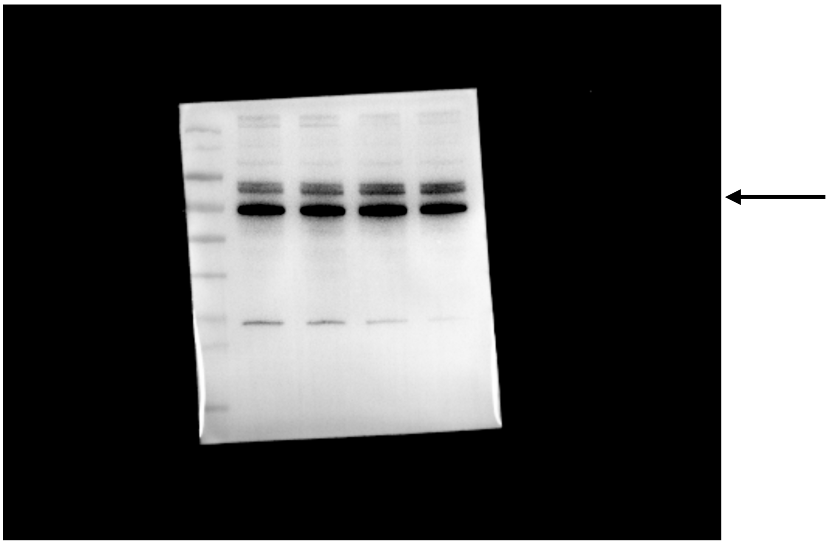

P 6 5for Figure 1E , the weight of molecular: 6 5 KDa
The order of sample (from left to right): Sham, ICH, ICH+CL218872, ICH+MRK-016

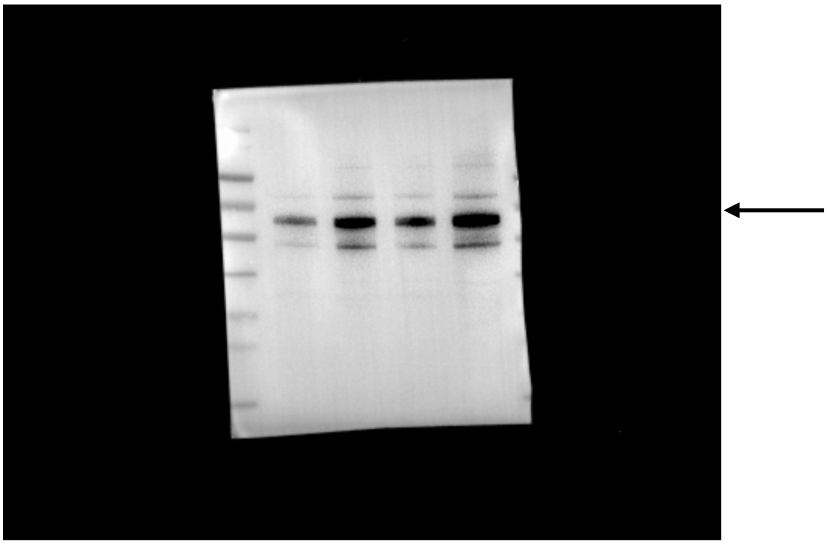

p - P 6 5 for Figure 1E, the weight of molecular: 6 5 KDa
The order of sample (from left to right): Sham, ICH, ICH+CL218872, ICH+MRK-016

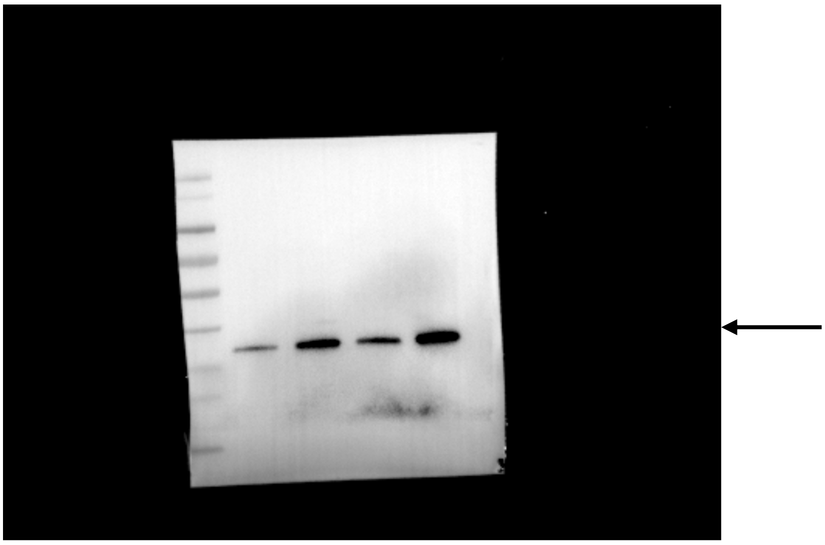

T N F - α for Figure 1E , the weight of molecular: 2 6 KDa
The order of sample (from left to right): Sham, ICH, ICH+CL218872, ICH+MRK-016

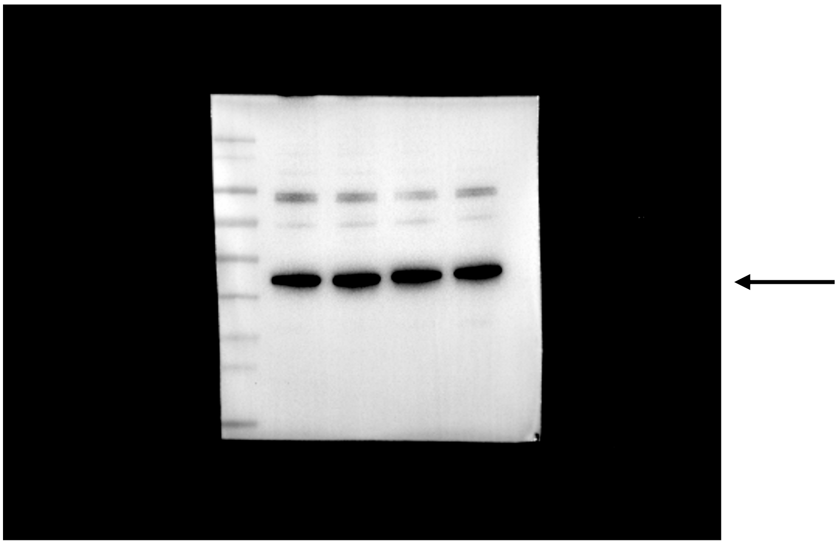

β - a c t i n for Figure 1E , the weight of molecular: 4 3 KDa
The order of sample (from left to right): Sham, ICH, ICH+CL218872, ICH+MRK-016

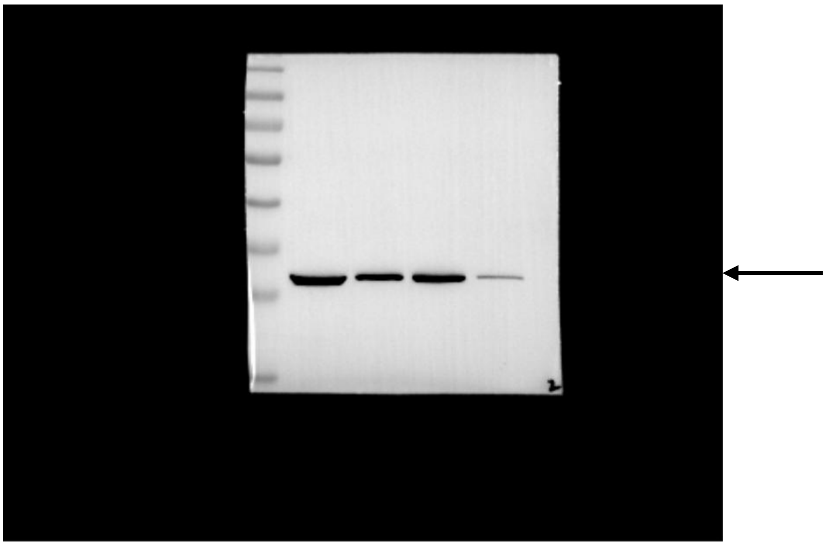

BDNF for Figure 5A; the weight of molecular: 28KDa

The order of sample (from left to right): Sham, ICH, ICH+CL218872, ICH+MRK-016

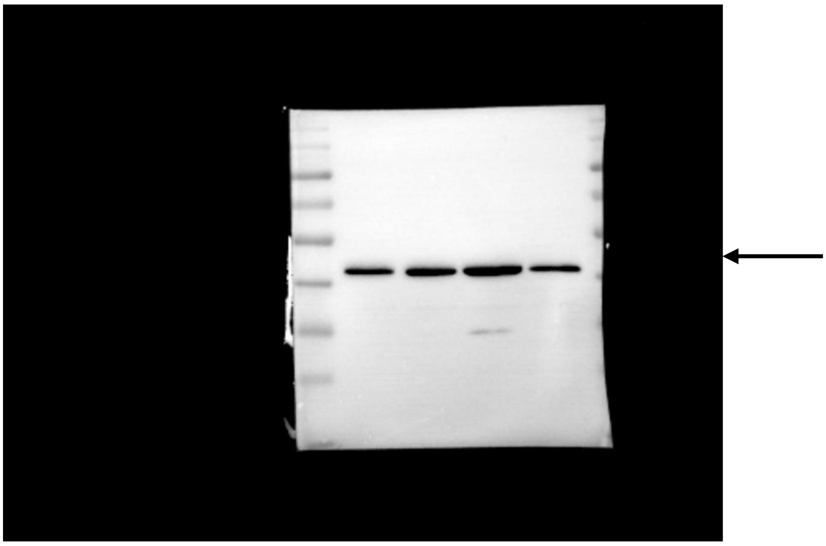

GAP43 for Figure 5A; the weight of molecular: 43KDa

The order of sample (from left to right): Sham, ICH, ICH+CL218872, ICH+MRK-016

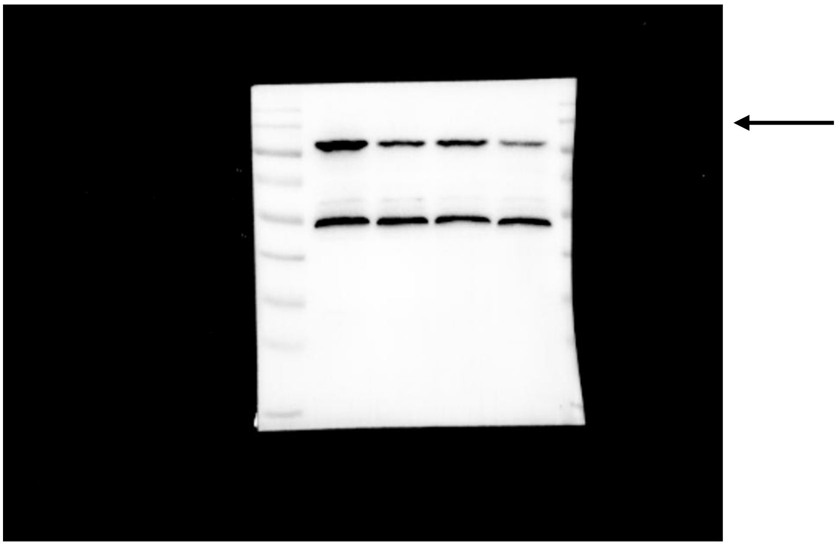

PSD95 for Figure 5A , the weight of molecular: 105KDa

The order of sample (from left to right): Sham, ICH, ICH+CL218872, ICH+MRK-016

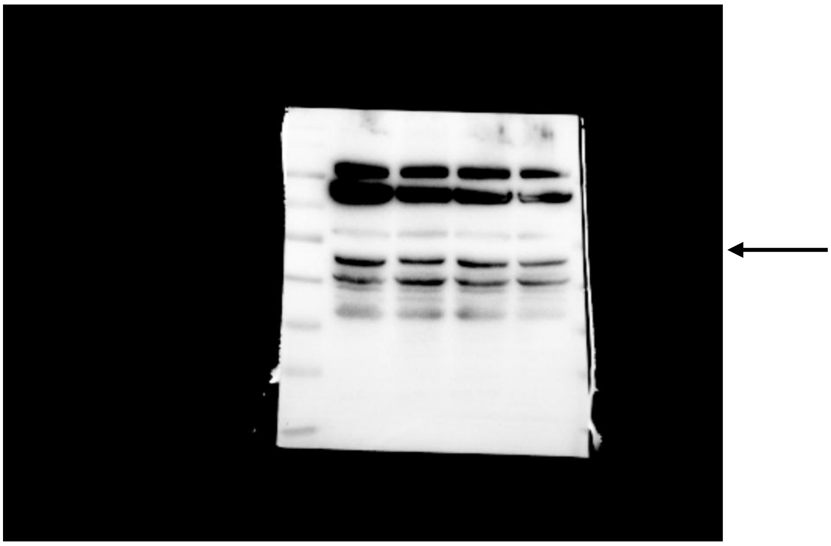

Synaptophysin for Figure 5A, the weight of molecular: 38KDa

The order of sample (from left to right): Sham, ICH, ICH+CL218872, ICH+MRK-016

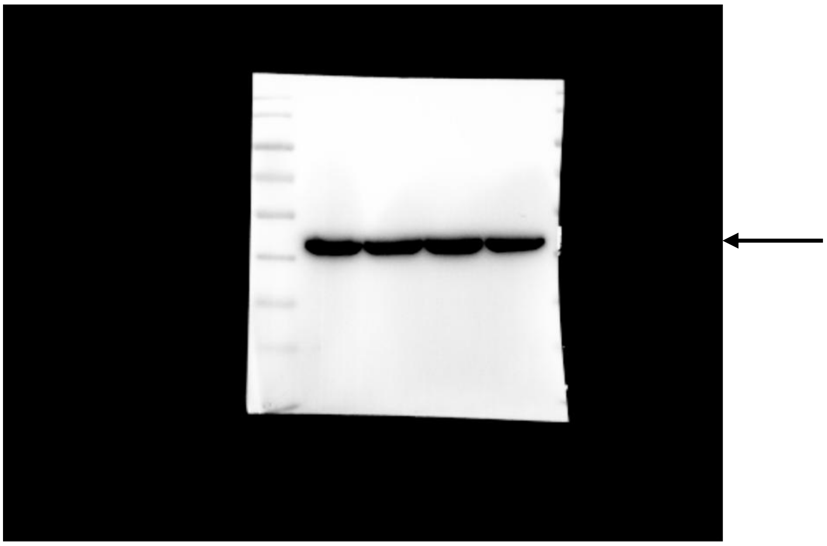

β-actin for Figure 5A, the weight of molecular: 43KDa

The order of sample (from left to right): Sham, ICH, ICH+CL218872, ICH+MRK-016
