# Supplementary material for: Effects of GABAAR modulators CL218872 and MRK-016 on neural repair and synaptic plasticity in mice with Intracerebral hemorrhage
Source: PLoS One. 2026 Mar 23;21(3):e0345025. doi: 10.1371/journal.pone.0345025 (PMC13008086; doi:10.1371/journal.pone.0345025)
Supplement: Attachment — Submitted filename: response letter.pdf [file pone.0345025.s008.pdf]

Dear Mr. Flores

Thank you for your email. The manuscript has been amended according to comments point by point.

1. Please ensure that you refer to Figure 4 in your text as, if accepted, production will need this reference to link the reader to the figure.

Response:

I’m sure Figure 4 is in the text, which in the line 356 of the page of 17 （clean copy.）

Best regards,

Yours,

Huang.
