# Supplementary material for: Effects of GABAAR modulators CL218872 and MRK-016 on neural repair and synaptic plasticity in mice with Intracerebral hemorrhage
Source: PLoS One. 2026 Mar 23;21(3):e0345025. doi: 10.1371/journal.pone.0345025 (PMC13008086; doi:10.1371/journal.pone.0345025)
Supplement: Attachment — Submitted filename: response to reviewer20250917.pdf [file pone.0345025.s009.pdf]

PONE-D-25-22559R2

Effects of GABAaR modulators CL218872 and MRK-016 on neural repair and synaptic plasticity in mice with Intracerebral hemorrhage

Professor Fei Huan

Editor’s comment:

1. "We note that your Data Availability Statement is currently as follows: "All data are in the manuscript and/or supporting information files" as your Data Availability Statement "

Please confirm at this time whether or not your submission contains all raw data required to replicate the results of your study. Authors must share the “minimal data set” for their submission. PLOS defines the minimal data set to consist of the data required to replicate all study findings reported in the article, as well as related metadata and methods (https://journals.plos.org/plosone/s/data-availability#loc-minimal-data-set-definition).

For example, authors should submit the following data:

- The values behind the means, standard deviations and other measures reported;
- The values used to build graphs;
- The points extracted from images for analysis.

Authors do not need to submit their entire data set if only a portion of the data was used in the reported study.

If your submission does not contain these data, please either upload them as Supporting Information files or deposit them to a stable, public repository and provide us with the relevant URLs, DOIs, or accession numbers. For a list of recommended repositories, please see https://journals.plos.org/plosone/s/recommended-repositories.

Response: Raw data file named as PONE-D-25-22559R2-2025-9-17, which has been uploaded in submission system as supporting information, meanwhile has been uploaded in German Neuroinformatics Node/G-Node (GIN) https://gin.g-node.org/FeiHuan/GABAaR/src/master/PONE-D-25-22559R2-2025-9-17.xlsx

kind regards,

Dr.Huan
