# Supplementary material for: Effects of GABAAR modulators CL218872 and MRK-016 on neural repair and synaptic plasticity in mice with Intracerebral hemorrhage
Source: PLoS One. 2026 Mar 23;21(3):e0345025. doi: 10.1371/journal.pone.0345025 (PMC13008086; doi:10.1371/journal.pone.0345025)
Supplement: Attachment — Submitted filename: response letter20251113.pdf [file pone.0345025.s010.pdf]

Response letter

Dear editor,

Subject:PLOS ONE: Edits requested on your submission PONE-D-25-22559R3

Thank you for your comments. The manuscript was revised according to your suggestion.

1. Please remove your figures/ from within your manuscript file, leaving only the individual TIFF/EPS image files. These will be automatically included in the reviewer's PDF

Response:

1. Figures were removed from the manuscript file.

I'm looking forward to receiving any news from you.

Sincerely yours,

Dr. Huan,

Nov.13th,2025
