# Supplementary material for: Effects of GABAAR modulators CL218872 and MRK-016 on neural repair and synaptic plasticity in mice with Intracerebral hemorrhage
Source: PLoS One. 2026 Mar 23;21(3):e0345025. doi: 10.1371/journal.pone.0345025 (PMC13008086; doi:10.1371/journal.pone.0345025)
Supplement: Attachment — Submitted filename: response to reviewers20251125.pdf [file pone.0345025.s011.pdf]

PONE-D-25-22559R3

Effects of GABAAR modulators CL218872 and MRK-016 on neural repair and synaptic plasticity in mice with Intracerebral hemorrhage

PLOS ONE

Dear Maidelyn R. Peregrin,

Thank you for your email.

The manuscript has been made an amendment according to the requirement in your mail dated Nov.24th,2025.

Reviewers' comment:

1. Your ethics statement should only appear in the Methods section of your manuscript. If your ethics statement is written in any section besides the Methods, please delete it from any other section.

Authors' response:

Ethic approval was deleted from line 773 to 775. Ethics statement only appear in the Methods section of the manuscript, which between line 169 to 172.

Kind regards,

Dr. Huang

Nov.25th,2025

Dear Uwe Rudolph,

Thank you for your comments, now the revised version of the manuscript that addresses the points raised during the review process were submitted as a separate file.

- A marked-up copy of your manuscript that highlights changes made to the original version. You should upload this as a separate file labeled 'Revised Manuscript with Track Changes'.

  Response：A separate file labeled 'Revised Manuscript with Track Changes' that highlights changes made to the original version was uploaded.

- An unmarked version of your revised paper without tracked changes. You should upload this as a separate file labeled 'Manuscript'.

  Response：A separate file labeled 'Manuscript' without tracked changes was uploaded.

- A rebuttal letter that responds to each point raised by the academic editor and reviewer(s). You should upload this letter as a separate file labeled 'Response to Reviewers'.

  Response：rebuttal letter from May,26th to Nov.,19th ,2025, as flows:

Additional Editor Comments:

The reviewers did an excellent job at pointing out multiple weaknesses and problems with this manuscript. What is required from the authors is not just a "major revision", I would call it a massive major revision. The Editorial Board Member concurs with the points that the reviewers are making. The authors have to address every single point raised by the reviewers.

In addition to the points raised by the reviewers, I would like to point out that the authors have to provide data from the literature on the binding affinities and efficacies of the compounds used at alpha1-containing GABAA receptors, alpha2-containing GABAA receptors, alpha3-containing GABAA receptors, and alpha5-containing GABAA receptors. For MRK-016, such data can be found in S. Maramai et al., J. Med. Chem. 2020, 63:3425-3446. It should be clear to the readers of the current manuscript how selective these compounds are for major GABAA receptor subtypes.

Moreover, I am surprised that the authors do not cite other papers where modulators of the GABAA receptor have been used to treat animal models of stroke, see, e.g., A.N. Clarkson et al., Nature 468;2010:305-309. The author just mentioned also published additional studies of this kind. Such studies should be cited and the results obtained in the current manuscript should be discussed considering this literature.

Response：We sincerely appreciate the meticulous and in-depth review of our manuscript by the reviewers, who have accurately pointed out several shortcomings and issues in the paper. We fully understand and agree with the views of the reviewers and members of the editorial board that the revision requirements this time are far more than a general "major revision" but rather a large-scale and thorough revision. We will respond to each specific comment put forward by the reviewers carefully and item by item. In addition, we have supplemented the research of S. Maramai, A.N. Clarkson, and others in the revised manuscript.

Review Comments to the Author

Reviewer #1: Summary:

In this manuscript, Chen et al. investigated the impacts of two GABAergic modulators, CL-218,872 and MRK-016 to enhance and reduce GABAAR-mediated transmission, respectively, in a mouse model of intracerebral hemorrhage (ICH). The authors posit that inhibiting synaptic excitability may reduce post-ICH excitotoxicity resulting from neuronal tissue damage and the release of glutamate throughout the extracellular space. Chen et al. investigated the impact of these GABAergic compounds on post-ICH recovery using behavioral, histological, and morphological methods. Broadly, treatment with CL-218,872, a compound that enhances GABAergic transmission, mitigated the deleterious impacts of ICH as seen via a reduction in total hemorrhage area and expression of pro-inflammatory markers, neurological behavioral

improvements, prevents neuronal death, and upregulates the expression of synaptic growth factors. These effects are not recapitulated by administration of MRK-016, a compound that reduces GABAergic inhibitory tone, and thus the authors conclude the author's hypothesis that upregulation of GABAergic transmission following ICH is a novel treatment strategy.

Unfortunately, due to multiple technical and analytical oversights and weaknesses, I am unable to recommend this manuscript for publication at this time.

Response：We fully acknowledge the reviewers' profound insights into the limitations of this study and are aware that the current manuscript has deficiencies in the depth of mechanistic explanation, data completeness, and literature support. We will conduct the following comprehensive revisions to the highest standards.

Major Comments:

1. It is not clear why the authors chose to investigate these specific GABAergic modulators (CL-218,872 and MRK-016) over the many other compounds available. This is critical to interpretation of the results. The binding affinities and efficacies of GABAergic drugs are highly variable (even among compounds that broadly up- or downregulate GABAergic transmission) owing to the varied composition of GABAARs, their multiple binding sites, and their heterogenous distribution throughout the brain. The introduction to this manuscript provides substantial amounts of background information on the GABAergic system and its component receptors, but any discussion regarding the pharmacology of CL-218,872 and MRK-016 is lacking, and there is no mention of their GABAAR selectivity (or any discussion of GABAAR subunits at all). Far more time is spent in the introduction discussing the function of GABABRs, which is not a major focus of this work, and would be better used to discuss these shortcomings.

Response：Thank you very much for your profound insights and valuable comments on the basis for compound selection in the manuscript. We fully understand and agree with the core issue you pointed out: we failed to clearly explain why CL-218,872 and MRK-016 were specifically chosen for the study among numerous GABAergic modulators, which is crucial for interpreting the experimental results. In the revised manuscript, we will significantly streamline the unnecessary discussions on $GABA_aRs$ to make room for emphasizing and supplementing the specific scientific basis for selecting CL-218,872 and MRK-016. We will elaborate on the known pharmacological properties of these two compounds (especially data on their binding affinity, efficacy, and selectivity for key $GABA_aR$ subunits) and discuss how these specificities are related to our study design and hypotheses.

2. As a consequence, CL-218,872 and MRK-016 are poorly characterized in this manuscript. CL-218,872 is a primarily GABAAR α1-preferring partial agonist, but not described as such, and is instead referred to using varied terms, including "activator" and "GABAAR agonist." Similarly, MRK-016 is an

α5-preferring negative allosteric modulator of GABAARs, but is described as an "inhibitor," and an "antagonist." MRK-016 is not a GABAAR antagonist. CL-218,872 and MRK-016 are presented as opposites, whereas the reality is more nuanced: these compounds mediate GABAergic transmission differently (α1-containing GABAARs have a greater role in phasic inhibition at the synapse whereas α5-containing receptors are extrasynaptic and primarily mediate inhibitory tone), and act on different neuronal populations based on subunit composition. This is an important consideration, as Figures 2 and 3 incorporate hippocampal imaging, a region with disproportionately high abundance of α5-containing GABAARs (Fritschy & Mohler, 1995).

Response: Thank you for pointing out the issues in the description of CL-218,872 and MRK-016, which is indeed an aspect that needs improvement in our manuscript. Following your suggestions, we will accurately revise the properties and functions of these two compounds: explicitly defining CL-218,872 as a "$GABA_aR$ α1-selective partial positive allosteric modulator (PAM)" and correcting MRK-016 to an "α5-selective negative allosteric modulator (NAM)". In addition, we will pay close attention to the hippocampal imaging issues mentioned in Figures 2 and 3, and cite the study by Fritschy & Mohler (1995) to support our arguments, thereby enhancing the rigor of the discussion.

3. Neuroanatomical information is either unclear or missing. It is not described where the TEM images in Figure 4 were taken (broadly mentioned as "brain tissue" in the legend), or if they are all from the same region. Similarly, Figures 2 and 3 are exclusively comprised of hippocampal imaging, but the rationale for choosing this region is not discussed. Figure 3 incorporates mean fluorescent intensity measurements, but it is not described how or where these measurements were made. The methods only describe "brain tissue," which is unclear and hinders interpretation of the quantification in Figures 3B and 3C. Notably, the word "hippocampus" is only used once, and it is in the discussion.

Response： Thank you for pointing out the issues regarding neuroanatomical information. During sample collection, we extracted the entire brain tissue, and for pathological analysis, we selected the bleeding site and regions where both $GABA_aR$ α1 and $GABA_aR$ α5 are expressed. That is to say, the images in Figures 2, 3, and 4 are from the hippocampal region. In addition, Image J was used to measure the average fluorescence intensity.

4. A large amount of the discussion section is used to re-introduce background information mentioned in the introduction (such as explaining the components of the GABAergic system) or describes the results. This would be better used to provide data interpretation or mechanistic evidence to strengthen the author's model to treating ICH via GABAergic modulation. For example, line 393 states "S44819, a GABAa α5 antagonist, enhances neurological recovery and peri-infarct brain remodeling through mechanisms of promoting the secretion of neurotrophic and angiogenesis-related factors" – this appears to contradictory to the author's findings, but is not discussed. Although not in an ICH model, α5-preferring negative allosteric modulators like MRK-016 are commonly reported in the literature to promote neuroplasticity and synaptic

strengthening (Fischell et al., 2015, Bugay et al., 2020, Troppoli et al., 2022), and this discrepancy should be addressed.

Response：Thank you for your valuable suggestions on the discussion section. We have removed much of the background information and focused on discussing the results obtained in this study as well as the contradictory points.

5. Benzodiazepines are commonly prescribed to treat psychiatric symptoms following stroke, but may impair recovery or worsen health outcomes (Torres et al., 2024, Colin et al., 2019, Goldstein, 1998). Therefore, promoting GABAergic activity in humans appears to have mixed utility, complicating the potential clinical role of CL-218,872, and should also be addressed in the discussion.

Response: Thank you for this important point raised by the reviewer. We have supplemented the relevant content in the discussion section.

6. The GFAP results are somewhat paradoxical. The authors describe the increased GFAP expression following CL-218,872 activation as indicative of "astrocyte activation, which may contribute to the amelioration of ICH-induced neuronal injury." However, increased GFAP expression is often used as a biomarker for inflammation-activated astrocytes. This is described in the discussion, where upregulation of GFAP is "demonstrated to be predictive of increased mortality and an adverse prognosis in patients with ICH," and appears to contradict the central hypothesis and earlier data interpretation and should be discussed further.

Response: Thank you for pointing out the contradictions and related issues in the GFAP results. We have recognized the importance of this contradiction for interpreting the research conclusions. We will conduct an in-depth discussion on this contradiction in the revised manuscript.

7. The antibody used for labeling GABAARs (AGA-016-200UL) targets γ1 subunits, but is described in the manuscript as a general marker for GABAAR expression (as in Figure 3), implying it labels all GABAARs. The most common GABAAR configuration incorporates γ2 subunits, not γ1, and thus this approach is unsuitable to support the conclusion of GABAAR upregulation. This should either be clarified, or a different labeling approach should be used.

Response: Thank you for pointing out the issue regarding the use of antibodies for $GABA_aR$ labeling. Due to the lack of specificity of the antibodies used, we will remove this section.

Minor Comments:

1. The authors make numerous references to the GABAC receptor—this is outdated nomenclature for this class, which should instead be referred to as the GABAA-rho receptor.

Response：Thank you for pointing out the issue with the receptor nomenclature. We have removed it.

2. Hemorrhage is spelled “haemorrhage” in the list of keywords, but not elsewhere.

Response：Thanks to the reviewers for pointing out the spelling problem in the keywords, which we have corrected.

3. It is not clear in the methods (line 159) how CL-218,872 or MRK-016 are administered.

Response：Thanks to the reviewers for pointing out the problems, the mode of administration, dosage and frequency have been added.

4. The neurological deficit scoring criteria should be further explained. Line 167: “Scores range from 0-28, with 0 being normal and higher scores indicating more severe neurologic impairment.” Please describe this in more detail. How was this scored? What is the maximum score per category?

Response：Thank you to the reviewers for pointing out the problem, and the rules for scoring neurologic deficits have been added.

5. Additionally, the neurological deficit score results are only broadly described as having an “improvement effect” or a “significant inhibitory effect” on “neurobiological function.” This is unclear, vague, and should be reworded. What behaviors were specifically impacted?

Response: Thanks to the reviewers for pointing out the problems, which have been revised.

6. It is not clear in the methods section how long after the ICH model the histology is performed. Given the timecourse of behavioral changes in Figure 1B, this is critical to include.

Response：Thanks to the reviewers for pointing out the problem, we took mouse brain tissue on the seventh day after treatment for pathology and molecular testing, which focused on the hippocampal region and molecular testing concentrated on the whole brain tissue.

7. Line 246: Capitalize Graphpad Prism and include the company/country. Additionally, consider including the F and degrees of freedom for ANOVA calculations in the results.

Response: Thank you for your comments, we have revised it.

8. Please clarify in the results section for Figure 1 that Figure 1C and 1D are derived from ELISA and Figure 1F, G and H are from western blotting.

Response：Thank you for your comments, which we have addressed in the illustration

9. The orientation of tissue in Figure 1A is inconsistent, with the 4th image in the ICH row and the 5th in the ICH+MRK-016 row upside-down (cortex facing downward).

Response：Thank you for your comments, we have revised it.

10. The Y-axis scale in Figure 1F for p65 expression ranges between 0.9 and 1.05, while it is between 0 and 1.5 for figures 1G and 1H.

Response：Thank you for your comments, we have revised it.

11. Figure 2A: The hippocampal inset for the sham treatment is reversed left-to-right.

Response：Thank you for your comments, we have revised it.

12. Figure 2C: The inset image for the Golgi staining is much smaller than the box depicts. The conclusions drawn from the Golgi staining in the ICH+CL-218,872 group are also somewhat unconvincing, as the dendritic morphology is difficult to determine and the overall neuronal density, especially in the overview image, appears lower than the MRK-016 group.

Response：Thank you for your comments, we have revised it.

13. Figure 3A: The fluorescent staining images between the ICH and ICH+MRK-016 groups are very similar. By eye, the example image for ICH+MRK-016 appears to have even greater GFAP reactivity than the ICH group, and does not appear to be significantly reduced as described in Figure 3C. This should either be re-analyzed, or another representative image should be chosen.

Response：Thank you for your comments, we re-selected the representative images and analyzed the fluorescence intensities using Image J.

14. Figure 4: The insets for each TEM image are not aligned with the outlined area, especially in the ICH group. The unbolded red text labels are also difficult to read, consider using a different color, bolding, or outlining strategy.

Response：Thank you for your comments, we have revised it.

15. Figure 4: The scale on the images is listed in nm while the legend lists it in μm.

Response：Thank you for your comments, we have revised it.

16. Line 341-346: There's mention of a significant increase in the number of synaptic junctions following CL-218,872, and a significant decrease in vesicles following MRK-016 administration, but these qualitative assessments are somewhat unclear. For example, the image for the ICH+MRK-016 group has 4 labeled vesicles while the ICH+CL-218,872 group only has 3.

Response：Thank you for your comments, we have revised it.

17. Line 355 has a typo: "…indicative of compromised synaptic in mice." Synaptic what?

Response：Thank you for your comments, we have revised it.

18. Line 360: There is mention that GAP-43 is significantly increased following ICH, and that this indicates "the repair mechanism of the organism has been activated… to promote synaptic remodeling." This is an interesting observation, but the conclusion is somewhat muddled by the reduction in other plasticity markers, and should be discussed.

Response：Thank you for your comments, which we have added to the discussion.

19. Line 383: It is a bit reductionist to say that GABA has a role in preventing insomnia and depression, as its utility in regulating synaptic excitability and synchronous activity applies to myriad CNS functions and psychiatric conditions. This should be either elaborated on or removed.

Response：Thank you for your comments, we have revised it.

20. Line 403: “Involved in several aspects of regulation, including memory, myopia, pain and sleep.” This is a sentence fragment.

Response：Thank you for your comments, we have revised it.

21. Line 404: “In the hippocampus, participates as an extrasynaptic receptor in the inhibition of neuronal excitability.” This sentence is also a sentence fragment.

Response：Thank you for your q comments, we have revised it.

22. Line 423-424: “which could decreased neuronal excitotoxicity.” Change to “decrease.”

Response：Thank you for your comments, we have revised it.

23. Line 430-434: “The administration of MRK-016 led to a further reduction in neuronal cell count and the appearance of intensified cytoplasmic staining, nuclear condensation, fragmentation, or lysis, suggesting that MRK-016 may inhibit the interaction between GABA and GABAaR, thereby exacerbating neuronal damage and apoptosis in ICH mice.” This states that, because MRK-016 enhances expression of inflammatory markers that are believed to be driven by a shift in the excitation:inhibitory balance, MRK-016 may inhibit GABAergic transmission. The ways in which MRK-016 alters GABAergic transmission have already been well-characterized through specific pharmacological studies (Atack et al., 2009), making the statement regarding binding interactions unnecessary.

Response：Thank you for your comments, which we have added to the discussion.

24. Line 457-459: “It is widely recognized that GABA promotes synaptic growth and neuroplasticity by activating voltage-gated calcium channels and N-methyl-D-aspartate (NMDA) receptors.” This should be reworded, as it implies GABA itself is an agonist at these receptors.

Response：Thank you for your comments, which we have added to the discussion.

25. Line 461-463: “These alterations were associated with prolonged transmission times for GABA from the presynaptic to the postsynaptic membrane, leading to impaired signal transduction.” This has no citation, and was not an experiment performed in this manuscript.

Response：Thank you for your comments, which we have added to the discussion.

26. Line 484-485: “…which indicated that the synaptic activity was reduced in the brain tissues of the rat model of ICH…” This is a mouse model of ICH.

Response：Thank you for your comments, which we have added to the discussion.

27. Line 490-493: “This study demonstrated that GABAaR agonist (CL218872) reduced the hemorrhage area, mitigates inflammatory responses, promotes the repair of damaged neurons, and enhances synaptic plasticity through the potentiation of interactions between GABA and GABAaR.” While it is probable that CL-218,872 upregulates GABAergic transmission, this manuscript does not directly link any neuroprotective effects post-ICH to changes in target engagement or the affinity of the GABAAR to GABA. Additionally, as CL-218,872 is believed to be a partial agonist, its primary pharmacological actions are likely not due to enhancing the receptor’s affinity to GABA.

Response：Thank you for your comments, which we have added to the discussion.

28. Many acronyms in the methods are not in the list of abbreviations. Line 138: SPF as an acronym is not mentioned again, and is also not in the list of abbreviations, as is HRP on line 241. Line 213: TEM is not in the list of abbreviations, etc.

Response：Thank you for your comments, which we have added to the discussion.

29. The abbreviation for SJ (synaptic junction) isn’t listed in the list of abbreviations. Similarly, when first listed on line 333, “SJ” seems to be listed as an abbreviation for “synapses” rather than synaptic junctions.

Response：Thank you for your comments, which we have added to the discussion.

30. Line 481: SYN as an acronym isn’t described and not in the list of abbreviations.

Response：Thank you for your comments, which we have added to the discussion.

31. Line 505: Reword to “There are no competing interests.”

Response：Thank you for your comments, which we have added to the discussion.

32. PD is misspelled as “pstsynaptic membrane” in the list of abbreviations.

Response：Thank you for your comments, which we have added to the discussion.

33. There are a number of errors with the formatting of the citations, particularly in the author’s names, which seem to be occasionally combined with the journal’s abbreviation. For example, line 601: Liu IYJTCMJ should be: Liu, I. Y. Line 612: Mewett KNJNr, should be: Mewett, K. N. Line 639: Bagetta GJCOiP should be: Bagetta, G, etc.

Response：Thank you for your comments, which we have added to the discussion.

34. Uncropped western blots should be made available as supplementary information.

Response：Thank you for your comments, which we have added to the discussion.

Reviewer #2:

This paper described studies in which the GABA-A positive allosteric modulator (PAM) CL218872 and the negative allosteric modulator (NAM) MRK-016 were evaluated for behavioral and cellular/molecular effects in mice with intracerebral hemorrhage (ICH)-induced neurotoxicity. ICH was induced in adult male C57BL/6J mice using a type IV collagenase infusion into the striatum, with sham surgery controls. Following recovery from surgery, the mice were treated with single doses of CL218872, MRK-016, or saline via gavage for 7 days (once/day). Across both behavioral and neurobiological measures, the overall finding was that CL218872, but not MRK-016, improved outcomes (behavioral, inflammatory, several standard markers of neuronal/synaptic integrity), leading the authors to conclude that CL218872 has therapeutic potential for reversing deficits associated with ICH.

This is a relatively straightforward study with mostly appropriate controls and a rigorous array of behavioral and cellular/molecular endpoints associated with ICH. While the results with CL218872 were modest and did not represent a full recovery, they were nevertheless interesting and encouraging. The manuscript is well prepared, and the conclusions justified by the results.

The manuscript needs some revising in order to better understand the results. A major omission by the authors was information on the GABA-A receptor selectivity of the two compounds chosen for study. Moreover, there was a tendency to be imprecise in describing the compounds, at different points using the terms “agonist”, “antagonist”, “activator”, “inhibitor” which would make the manuscript difficult to understand to those unfamiliar with these compounds. I recommend referring to CL218872 as a PAM and MRK-016 as a NAM. It’s also important to identify CL218872 to a “partial” PAM, as it has intrinsic efficacy less than a full PAM. Note that on page 8, line 146, MRK-016 is incorrectly labeled as an antagonist.

The manuscript would also benefit from much more information on why the authors chose these two compounds, and even if it was not due to GABA-A subtype selectivity, the potential implications for having regenerative effects of an alpha-1 subtype-selective compound should be discussed. Likewise, discussion regarding the alpha-5 subtype selectivity for MRK-016 should be discussed. Since these studies were with single doses only, some discussion regarding how these doses were chosen and, if possible, the extent to which they relate to occupancy of relevant receptors/receptor subtype would be helpful. Also, why were they administered once per day for 7 days? As a minor point, I could not find a description of the vehicles used for these two drugs.

Regarding methodology, some more detail about the behavioral procedures would be helpful. Were the observers unaware of the treatment conditions? Was more than one observer used, and if so, how were the data handled? Unfortunately, the study design did not permit assessment of the effects of the two compounds in sham animals—do the authors have any information regarding effects that might show up with their rating scales in non-ICH mice?

Response: We would like to thank reviewer 2 for his recognition of this study and his valuable suggestions, which we will improve accordingly. Regarding the compound description, we will add information on the GABA-A receptor selectivity of CL218872 and MRK-016, and standardize the terminology to Positive Alteration Modulators (PAM) and Negative Alteration Modulators (NAM) to make it clear that CL218872 is a partial PAM. With respect to compound selection, we elaborate on its consideration based on GABA-A subtype selectivity (CL218872 prefers the α1 subtype, MRK-016 prefers the α5 subtype) and add a discussion of the regenerative effect potential of α1 subtype-selective compounds and the impact of α5 subtype selectivity on the action of MRK-016. In the dosage selection section, we will clarify that the dosage was determined with reference to previous studies and pre-tests, and will add receptor occupancy data, if available; the basis for the frequency of administration (once a day for 7 days) will be explained in the context of the pathologic progression of ICH and pharmacokinetic characteristics, and we will add specific information on the carriers used for the two drugs. In the Behavioral Methods section, we will specify that observers were assessed in a blinded manner, and will add methods for combining data if multiple observers were used.

Minor corrections:

Page 17, line 355: word(s) missing in this sentence

Response: Thank you for your comments, we have revised it.

Page 19, line 383: “increased GABA levels”...should this be “decreased GABA level”?

Response: Thank you for your comments, we have revised it.

Page 19, line 393: This is the first place GABA-A subtypes are mentioned. Also, S44819 is an unusual compound, in that it may not bind to the benzodiazepine site. A bit more information and context with this compound is recommended.

Response: Thank you for your comments, we have revised it.

Page 20, lines 404-405: Sentence beginning with “In the hippocampus...” words are missing.

Response: Thank you for your comments, we have revised it.

Page 20, line 420: “levels of both” but 3 items are listed (“both” could be removed)

Response: Thank you for your comments, we have revised it.

Page 22, lines 453-459: The relevance of the discussion of GABA’s unique role in development to the present study is unclear and needs more development. Action of GABA at NMDA receptors and calcium channels is, to my knowledge, unique to development and not necessarily playing a role in plasticity for any type of neuronal growth or repair in adulthood (again, to my knowledge—it’s not my area of expertise). Maybe this section isn’t needed, since it seems to go beyond the scope of the studies?

Response: Thank you for your comments, we have revised it.

Dr.Huan,

July,28th,2025

Dear Mr. Flores

Thank you for your email. The manuscript has been amended according to comments point by point.

1. Please ensure that you refer to Figure 4 in your text as, if accepted, production will need this reference to link the reader to the figure.

Response:

I’m sure Figure 4 is in the text, which in the line 356 of the page of 17 （clean copy.）

Dr.,Huan,

Aug.,15th,2025

Dear editors,

Thank you for your email.

PONE-D-25-22559R2

Effects of GABAaR modulators CL218872 and MRK-016 on neural repair and synaptic plasticity in mice with Intracerebral hemorrhage

We have made revised point-by-point to your comments. Details as flows,

1. We notice that your revision was submitted on Aug 28 2025 , but the manuscript file in your submission's file inventory was uploaded on Aug 14 2025. Please upload the latest version of your revised manuscript as the main article file, with the item type 'Manuscript,' ensuring that it does not contain any tracked changes or highlighting. This will be used in the production process if your manuscript is accepted.

Response： the latest version of revised manuscript has been uploaded as the main article file, and not contain any tracked changes or highlighting.

2. Thank you for stating the following financial disclosure:

"Science and Technology Project of Health Commission of Sichuan Province (Project No. 23LCYJ007)."

Please state what role the funders took in the study. If the funders had no role, please state: "The funders had no role in study design, data collection and analysis, decision to publish, or preparation of the manuscript."

If this statement is not correct you must amend it as needed.

Please include this amended Role of Funder statement in your cover letter; we will change the online submission form on your behalf.

Response: The funders had no role in study design, data collection and analysis, decision to publish, or preparation of the manuscript.

3. Please note that funding information should not appear in the Acknowledgments section/Funding section or Any other areas of your Manuscript. We will only publish funding information present in the Funding Statement section of the online submission form. Please remove any funding-related text from the manuscript.

Response: Any funding-related text has been removed from the manuscript.

4. Will you please specify where in your manuscript is you data sharing.

Response: This study did not generate any new datasets. All data analyzed are from publicly available sources, as cited in the manuscript.

5. Can you please upload an additional copy of your revised manuscript that does not contain any tracked changes or highlighting as your main article file. This will be used in the production process if your manuscript is accepted. Please amend the file type for the file showing your changes to Revised Manuscript w/tracked changes. Please follow this link for more information: http://blogs.PLOS.org/everyone/2011/05/10/how-to-submit-your-revised-manuscript/

Response: An additional copy of your revised manuscript that does not contain any tracked changes or highlighting as the main article file has been uploaded.

6. Please remove your figures/ from within your manuscript file, leaving only the individual TIFF/EPS image files. These will be automatically included in the reviewer's PDF

Response: All figures has been removed from manuscript file.

7. Please provide additional details regarding participant consent. In the Methods section, please ensure that you have specified (1) whether consent was informed and (2) what type you obtained (for instance, written or verbal). If your study included minors, state whether you obtained consent from parents or guardians. If the need for consent was waived by the ethics committee, please include this information.

Response: Participant consent is not applicable. All procedures of animal experiment were performed according to the Guide for the Care and Use of Laboratory Animals of the National Institutes of Health and were approved by the Yunnan Beisitai Biotechnology Co., Ltd (BST-MICE-20240127-01).

Dr.,Huan

Sep.3th,2025

Editor’s comment:

1. "We note that your Data Availability Statement is currently as follows: "All data are in the manuscript and/or supporting information files" as your Data Availability Statement "

Please confirm at this time whether or not your submission contains all raw data required to replicate the results of your study. Authors must share the “minimal data set” for their submission. PLOS defines the minimal data set to consist of the data required to replicate all study findings reported in the article, as well as related metadata and methods (https://journals.plos.org/plosone/s/data-availability#loc-minimal-data-set-definition).

For example, authors should submit the following data:

- The values behind the means, standard deviations and other measures reported;

- The values used to build graphs;

- The points extracted from images for analysis.

Authors do not need to submit their entire data set if only a portion of the data was used in the reported study.

If your submission does not contain these data, please either upload them as Supporting Information files or deposit them to a stable, public repository and provide us with the relevant URLs, DOIs, or accession numbers. For a list of recommended repositories, please see https://journals.plos.org/plosone/s/recommended-repositories.

Response: Raw data file named as PONE-D-25-22559R2-2025-9-17, which has been uploaded in submission system as supporting information, meanwhile has been uploaded in German Neuroinformatics Node/G-Node (GIN)

https://gin.g-node.org/FeiHuan/GABAaR/src/master/PONE-D-25-22559R2-2025-9-17.xlsx

kind regards,

Dr.Huan

Sep.,17th, 2025

Reviewer #1: Chen et al. have significantly revised this manuscript to address many of the points raised in my first review, such as incorporating information regarding the mechanisms of both CL-218,872 and MRK-016, providing raw data and expanding the description of the methods employed. These changes have notably improved the quality of the manuscript. However, I believe further revisions are necessary, especially in the discussion, with a focus on improving accuracy, the putative mechanisms of these compounds, and how the author’s findings agree or disagree with the other cited works from the literature.

Response： We sincerely thank the Reviewer for their positive assessment of our revisions and for acknowledging the improvements made to the manuscript. We also appreciate the Reviewer's valuable suggestions for further enhancing the discussion. Following these recommendations, we have thoroughly revised the Discussion section to improve accuracy, elaborate on the putative mechanisms of CL-218,872 and MRK-016, and more clearly articulate how our findings align or contrast with existing literature.

Major points:

1) The authors now discuss the heterogenous subunit composition of GABAARs and the primary binding affinities for both CL-218,872 (primarily an alpha1 PAM) and MRK-016 (primarily an alpha5 NAM) in the introduction. However, they should still reference the full binding profile of these compounds at other GABAAR subunits as previously requested by the editor. Both of these GABA modulators have non-negligible effects at different receptor populations with unique localization throughout other brain structures that are also likely impacted by ICH. This would further strengthen the manuscript and interpretation of results.

Response: We sincerely thank the reviewer for this critical suggestion. We fully agree that clarifying the full binding profiles of both compounds at other GABAaR subunits is essential for a comprehensive understanding of their pharmacological actions and our results. As suggested, we have now supplemented the full binding profiles of both CL-218,872 and MRK-016 in the Introduction section.

Additionally, much of the histology employed in this manuscript is within the hippocampus. Chen et al. discuss the prevalence of the alpha5 subunit in hippocampal structures, which is mechanistically relevant

to the primary binding affinity of MRK-016. However, in the introduction, the authors state the alpha1 subunit (CL-218,872's target) is "widely expressed in the thalamus, cortex, and basal ganglia." Without referencing the expression of these receptors within the hippocampus, it is difficult to interpret how CL-218,872 is acting to improve hippocampal ICH outcomes.

Response: We thank the reviewer for this insightful observation. The point raised is indeed critical, as our previous introduction did not adequately address the expression of the α1 subunit in the hippocampus to mechanistically explain the hippocampal benefits of CL-218,872. To address this gap and provide a clear mechanistic rationale, we have made an important addition to the Introduction.

2) Some of the newly-provided binding information provided for CL-218,872 and MRK-016 is incorrect. Lines 122-126 state these compounds bind at the "$\alpha1^+$ /β^- " and "$\alpha5^+$ /β^- " interface, respectively. However, the binding location for both compounds is at the benzodiazepine-binding site, comprised of an alpha and a gamma subunit, not a beta subunit.

Response: We sincerely thank the reviewer for correcting this important pharmacological inaccuracy. We have now corrected the text to accurately reflect that both CL218872 and MRK-016 bind to the classical benzodiazepine site at the interface between the respective alpha subunit (α1 or α5) and the γ subunit.

3) The authors should provide additional rationale for their focus on the hippocampus in the introduction and discussion and its relevance to clinical pathology. They state that their focus is because their preclinical ICH model induces "concentration of hemorrhage in hippocampal-adjacent" regions and "high hippocampal expression of GABAaR α1 and α5." However, it is not made apparent how or if the hippocampus is uniquely vulnerable to ICH-induced injury in human cases, and why targeting this structure specifically may improve overall behavioral treatment outcomes.

Response: We sincerely thank the reviewer for this insightful comment. We agree that providing a stronger rationale for our hippocampal focus is crucial for contextualizing our findings. In the revised manuscript, we have expanded our introduction and discussion to address this point more comprehensively.

4) The authors have improved their discussion of the increase in GFAP expression following CL-218,872 administration, indicating that it may be indicative of neuroprotection and not increased inflammation. However, they state that CL-218,872 may produce a "shift in astrocytes towards a beneficial phenotype" that "likely represents a significant component of its neuroprotective mechanism." It is unclear how

CL-218,872 may produce this change in astrocytic activity, and therefore appears somewhat speculative. As the authors state that this is likely a critical aspect of the underlying neuroprotective mechanism, this should be expanded upon. For example, does CL-218,872 alter astrocyte activity via suppression of extrasynaptic glutamate release or alteration of the local E/I balance? Would CL-218,872 act directly upon astrocytes?

Response: We thank the reviewer for raising this critical point. We agree that elucidating the potential mechanism by which CL218872 influences astrocyte phenotype is essential. In the revised discussion, we have expanded on this aspect to provide a more substantiated and less speculative explanation

5) In the previous review, I stated: "Benzodiazepines are commonly prescribed to treat psychiatric symptoms following stroke, but may impair recovery or worsen health outcomes (Torres et al., 2024, Colin et al., 2019, Goldstein, 1998). Therefore, promoting GABAergic activity in humans appears to have mixed utility, complicating the potential clinical role of CL-218,872, and should also be addressed in the discussion." While the authors have referenced its benzodiazepine-like side effects in the discussion such as "central nervous system depression, cognitive impairment, drug dependence and withdrawal symptoms," these off-target effects broadly hold true for the use of benzodiazepines in any context and do not fully address the point raised. The authors should more thoroughly address the mixed clinical literature regarding benzodiazepine use (e.g. generalized increase in GABAergic transmission and shift of the E/I balance) in ischemic or hemorrhagic brain injury and discuss how CL-218,872 administration (or localized GABAergic potentiation at alpha1-GABAARs) avoids these current treatment pitfalls.

Response: We are grateful to the reviewer for this insightful comment and for the opportunity to provide a more nuanced discussion on this critical translational issue. We agree that simply listing the general side effects of benzodiazepines is insufficient. In the revised manuscript, we have substantially expanded our discussion to directly engage with the mixed clinical literature on benzodiazepines post-stroke and to articulate the potential mechanistic distinctions of a subunit-selective agent like CL-218,872.

6) In the discussion, Chen et al. have now introduced the discrepancy in the literature between the neuroprotective and synaptic strengthening properties of MRK-016 in ischemic stroke vs. their findings that it exacerbates post-ICH injury. However, the points raised are not very detailed and seem primarily speculative. This would be strengthened in a number of ways, such as discussing how ICH is different than ischemic brain injury, which is only broadly described as having a "distinct pathological context." Other factors are briefly proposed such as "microenvironment, therapeutic time window, receptor activation state, MRK-016 dosage, and the degree of inhibition," but are not further discussed. How would these factors contribute to the paradoxical effects of MRK-016? Some of the points raised are easily addressable, e.g., is the dosage/administration route of MRK-016 uniquely different in this work compared to the ischemic

stroke literature? Others may require further consideration: what changes in the microenvironment are expected to play a role? Astrocytic involvement is raised as a contributory mechanism for the effects of CL-218,872, but not discussed here re: MRK-016.

Response: We sincerely thank the reviewer for this excellent and constructive feedback. We agree that our initial discussion regarding the discrepant effects of MRK-016 was underdeveloped. In the revised manuscript, we have substantially expanded this section

Minor points:

1) Line 22: “ICH mice were intervened by CL218872 and MRK-016 of GABAaR.” This is grammatically unclear and incorrect, reword to “ICH mice were administered the GABAAR modulators CL218872 or MRK-016,” or something similar.

Response: We thank the reviewer for pointing out the inappropriate phrasing. We fully accept this suggestion and have revised the sentence as recommended to: “ICH mice were administered the GABAAR modulators CL218872 or MRK-016.”

2) Line 38: “molecules were detected to assess” – change to “molecules were quantified to assess”

Response: We agree with the reviewer that "quantified" more precisely describes our work involving quantitative analysis than "detected." We have accordingly changed "detected" to "quantified" in the text.

3) Abstract, results: consider mentioning the results of MRK-016 in the abstract as well, as it does not mention the effects of MRK-016 anywhere.

Response: We thank the reviewer for this important reminder. We fully agree that the abstract should present the results for both compounds, CL218872 and MRK-016, to ensure completeness and comparability. We have now supplemented the results section of the abstract with the key findings concerning MRK-016.

4) Line 89, introductory paragraph: “directly impairs GABAergic neurons” – consider briefly discussing effects on glutamatergic neurons or rewording this, as it implies GABAergic neurons are the predominant casualty of ICH and excitotoxicity, which I don’t believe to be the case.

Response：We sincerely thank the reviewer for raising this important point. We fully agree that excitotoxicity affects various neuronal types in ICH, rather than specifically or predominantly impairing GABAergic neurons. Our original phrasing was indeed imprecise and potentially misleading. As suggested, we have revised the introductory paragraph to shift the focus from "impairing GABAergic neurons" to "disrupting GABAergic signaling," which more accurately captures the essence of the pathological process. The revised text now reads: "However, in the pathological milieu of ICH, excitotoxicity leads to a pervasive disruption of neuronal circuits. This disruption impacts GABAergic signaling, impairing the function of GABAergic synapses, and disturbing GABA synthesis, release, and reuptake. The net result is a significant attenuation of endogenous inhibitory tone."

5) Lines 145 and 146: CL-218,872 is incorrectly referred to as a GABAa receptor agonist (instead of a PAM), and MRK-016 is incorrectly called a GABAa receptor antagonist (instead of a NAM).

Response: We sincerely thank the reviewer for correcting this important pharmacological inaccuracy. We fully accept the criticism and apologize for the previous improper terminology. We have strictly revised the text on lines 145-146 as suggested.

6) Line 150: “sterically” should be “stereotactically.”

Response: We thank the reviewer for pointing out this error. The word "sterically" has been corrected to "stereotactically" in the manuscript.

7) Line 160: Please clarify the dilution of DMSO, or state that it is 100% DMSO.

Response: We sincerely thank the reviewer for raising this important point. The issues you highlighted are absolutely valid—the original text indeed omitted the specification of DMSO concentration and contained serious ambiguity in the description of dosing for the experimental groups, which undoubtedly compromised the clarity and reproducibility of the methodology. We have revised the manuscript accordingly as suggested. The revised version reads as follows: Pharmacological intervention commenced 24 hours postoperatively. Mice in the Sham and ICH groups received 200 μL of 1% DMSO in saline daily

via gavage; mice in the ICH+CL218872 group were administered a CL218872 solution (5 mg/kg, dissolved in 1% DMSO saline) daily via gavage; mice in the ICH+MRK-016 group were administered an MRK-016 solution (5 mg/kg, dissolved in 1% DMSO saline) daily via gavage. The administration was performed once daily for 7 consecutive days.

8) Line 162: "The CL218872 were provided…" change to "CL218872 was provided/administered"

Response: We thank the reviewer for pointing out the grammatical and wording inaccuracies. We have revised the sentence.

9) Line 212: "…incubated with species-appropriate secondary anti-IgG antibodies..." Please include details on the specific secondary antibodies used.

Response: We thank the reviewer for this suggestion. We have now included the specific details of the fluorescently-labeled antibody used for GFAP detection in the revised manuscript. The revised text now reads: Brain tissues were fixed with 4% paraformaldehyde (PFA) overnight at 4°C. Subsequently, 5 μm sections were prepared and washed with phosphate-buffered saline (PBS). After blocking with 10% goat serum containing 0.3% Triton X-100 for 1 hour at room temperature, the sections were incubated with the fluorescently-labeled primary antibody iFluor™ 488 Conjugated GFAP Mouse Monoclonal Antibody (1: 200 dilution) overnight at 4°C. Following PBS washes, nuclei were counterstained with 4′,6-diamidino-2-phenylindole (DAPI) (D9542, Sigma-Aldrich, USA). Three representative sections from each sample were examined under a fluorescence microscope (Leica, Germany). The fluorescence intensity of GFAP staining in the hippocampal regions was quantified using ImageJ software.

10) Figure 1A: It is difficult to determine structure or slice orientation (is it upside down?) in the last image provided in the ICH row. If possible, a better image should be provided.

Response: We thank the reviewer for this observation. We agree that the original image was not optimal for assessing the brain structure. We have now replaced the image in Figure 1A (ICH row) with a clearer graph that better shows the anatomical structure and correct orientation of the brain slice.

11) Figure 1C, 1E: MRK-016 is incorrectly written as "MRK-06."

Response: We sincerely thank the reviewer for their meticulous attention to detail in identifying this typographical error. We have now carefully checked and corrected the labels in Figures 1C and 1E, changing "MRK-06" to the correct "MRK-016" throughout.

12) When describing results between lines 271-280, a single reference to “figure 1C-E” is made at the end. This makes it difficult to link these descriptions back to an individual figure, especially given that some data are derived from Western blotting and some is from ELISA. It would be better to state which data are from Figure 1C, figure 1D, and figure 1E specifically.

Response: We thank the reviewer for this excellent suggestion to improve the clarity of our results description. We fully agree that explicitly linking each specific finding to its corresponding figure panel will significantly enhance the clarity and readability of the text. We have revised the results description in lines 271-280 as suggested.

13) Figure 2A: the inset for the sham group is vertically inverted compared to the full image.

Response: We sincerely thank the reviewer for their meticulous observation. The point raised is absolutely valid; the inset for the sham group in Figure 2A was indeed vertically inverted compared to the full image. We have reprocessed the image to ensure the orientation of the inset is consistent with the main panel and have updated the figure accordingly.

14) Figure 2B: the insets appear poorly aligned with the full image in the ICH and ICH+CL-218,872 group, and especially in the ICH+MRK-016 group, where it is difficult to determine which hippocampal structure is actually in the inset.

Response: We appreciate your input regarding Figure 2B and have revised the figure to address your concern.

15) Figure 2C: the inset for the ICH group appears horizontally inverted compared to the full image.

Response: We appreciate your input regarding Figure 2C and have revised the figure to address your concern.

16) Line 291: “H&E staining revealed The ICH group…” change T to lowercase (“revealed the ICH group”).

Response: We thank the reviewer for pointing out this typographical error. We have corrected "The" to "the" at the beginning of the sentence on line 291 to ensure grammatical accuracy. The manuscript has been updated accordingly.

17) Line 321: “n=5 biologically independent samples per group” is unclear and should be reworded to n=5 animals per group, or something similar.

Response: We thank the reviewer for highlighting the lack of clarity in this phrasing. We have accepted the suggestion and revised the text from "n=5 biologically independent samples per group" to "n=5 mice per group" to accurately reflect the experimental design.

18) Line 326/327: “strikingly, CL218872 treatment elevated GFAP expression to near-sham levels.” As figure 3B still does appear to show a decrease in GFAP expression after treatment over sham surgery alone, please include either average GFAP expression intensity for both of groups or the p-value comparison (or both).

Response: We are grateful to the reviewer for prompting us to clarify this point. We agree that a precise statistical comparison is essential. Upon re-analysis, the direct comparison between the CL-218872 group and the Sham group did yield a statistically significant increase ($p < 0.05$), which we have now clearly stated in the revised manuscript. We have modified the text as follows: "In contrast, CL-218872 treatment significantly elevated GFAP expression above the level of the Sham group ($p < 0.05$)."

19) Line 355: “(n=3)” – change to “n=3 animals.”

Response: We thank the reviewer for suggesting greater clarity on this point. We have revised the text by changing "(n=3)" to "n=3 animals"

20) Figure 5 legend: Also include the n’s (presumably n=3 animals as well).

Response: We thank the reviewer for prompting the inclusion of this important information. We have updated the Figure 5 legend to explicitly state the sample size as "n=3 animals".

21) Line 360: "…were significantly reduced (p<0.001)" BDNF was reduced at p<0.05 level, while PSD95 and synaptophysin were reduced at p<0.001. Either refer to the changes collectively as p<0.05, or list the p-values for each comparison, e.g. "expression levels of BDNF (p<0.05), PSD-95 (p<0.001) and synaptophysin (p<0.001) were significantly reduced."

Response: We sincerely thank the reviewer for pointing out this imprecise wording. We have revised the text to clearly indicate that the described changes were statistically significant ($p < 0.05$).

22) Line 365: (*p* < 0.05): remove the extra *.

Response: We thank the reviewer for catching this formatting error. We have removed the extra asterisk and corrected the text.

23) Line 370: "figure 5: CL218872 enhanced synaptic reconstruction" – more accurate to say "enhanced markers of synaptic reconstruction" (or formation/plasticity).

Response: We thank the reviewer for this important correction. We have revised the text to state "enhanced markers of synaptic plasticity".

24) Line 375: "Intracerebral Hemorrhage…" lower case h, "Intracerebral hemorrhage…"

Response: We thank the reviewer for pointing out this formatting issue. We have revised the text by changing "Intracerebral Hemorrhage" to "Intracerebral hemorrhage" as suggested.

25) Line 380: add a space after "mouse model of ICH." before "GABA…"

Response: We thank the reviewer for pointing out this formatting detail. We have added a space after "mouse model of ICH." before "GABA..." as suggested.

26) Line 383: “pathologically elevated GABA levels following ICH can induce neuronal hyperexcitability and excitotoxic injury.” This is incorrect, and is likely either erroneously referring to GABA instead of glutamate.

Response: We sincerely thank the reviewer for correcting this critical error in neuropharmacology. The sentence has been corrected by replacing "GABA" with "glutamate" and "GABAergic" with "excitatory-inhibitory" to accurately reflect the underlying mechanism.

27) Line 385: “precise modulation of the imbalanced GABAergic signaling pathway…” the introduction of the paper talks about supraphysiological glutamate and excitotoxic injury, then posits enhancing GABAergic transmission is a putative treatment. Thus, it would be more accurate to discuss this in terms of an imbalance between excitatory and inhibitory transmission, or clarify that this is referring to excitotoxicity that specifically impacts GABAergic neurons.

Response: We sincerely thank the reviewer for this insightful comment. We fully agree that framing the therapeutic strategy in terms of restoring the overall balance between excitatory and inhibitory transmission is more logical and accurate than focusing solely on the GABAergic pathway. As suggested, we have revised the text around line 385 to reframe our hypothesis in terms of "restoring the excitatory-inhibitory (E/I) balance," which more precisely connects the previous discussion on excitotoxicity with our proposed approach of GABAergic modulation.

28) Line 388: “the alpha1 subunit is the most widely distributed and functionally critical…” While it may be the most abundant subunit, it is inappropriate to say it is the most functionally critical.

Response: We thank the reviewer for this precise correction. We agree that describing the alpha1 subunit as the "most functionally critical" is an overstatement, as other subunits have distinct and vital roles. We have revised the sentence to state that it is "the most widely distributed and abundant subunit," which is more accurate and supported by the literature.

29) Line 390: “and is essential for maintaining CNS excitation-inhibition balance.” It’s involved in the E/I balance, but so are all the other subunits in different contexts. This should be reworded.

Response: We thank the reviewer for this valuable clarification. We agree that the E/I balance is a system-level function involving all GABA_A receptor subunits. We have reworded the sentence to more accurately reflect the role of the α1 subunit, stating that it "contributes significantly to maintaining CNS excitation-inhibition balance."

30) Line 391: "As a major component of the α1β2γ2 subtype, the α1 subunit mediates…" Each included subunit is a "major component" of the α1β2γ2 subtype. Additionally, introducing α1β2γ2 GABAARs here without the context that they're the most abundant subtype makes their relevance unclear.

Response: We thank the reviewer for these insightful comments. We have revised the text to address both points: (1) we have removed the problematic "major component" phrasing and instead clarify the functional role of the α1 subunit within the complex, and (2) we now explicitly state that the α1β2γ2 is the most abundant receptor subtype to provide essential context for its relevance.

31) Line 394: "this subunit is a key target for benzodiazepines," generally, classical benzodiazepines bind a range of alpha subunits (especially alpha2 and alpha3) on the GABAAR with relatively comparable affinity. As it is written, it implies benzodiazepines have unique selectivity for alpha1 subunits—consider rewording this to clarify.

Response: We thank the reviewer for correcting this inaccurate statement. We fully agree that classical benzodiazepines bind to a range of alpha subunits (e.g., α1, α2, α3, α5) with comparable affinity rather than showing unique selectivity for the α1 subunit. We have revised the text to accurately reflect their pharmacological profile.

32) Line 394: "its activation enhances chloride influx" – This is true of GABA-PAMs at every BZ-sensitive GABAAR subtype, is redundant with the previous sentence, and would best be removed.

Response: We thank the reviewer for pointing out the redundancy here. We agree that "enhancing chloride influx" is a general mechanism shared by all benzodiazepine-positive allosteric modulators and is covered in the preceding context. We have therefore removed this redundant phrase as suggested.

33) Line 395: “[alpha1] participates in regulating synaptic plasticity associated with learning and memory in the hippocampus.” – Yes, alpha1-containing GABAARs contribute to the E/I balance and thus plasticity (as do all GABAARs), but alpha5-containing GABAARs regulating tonic inhibition are generally considered to play a greater role in regulating hippocampal learning and memory.

Response: We thank the reviewer for this important clarification and for highlighting the more prominent role of α5-containing GABAARs in hippocampal-dependent learning and memory. We have revised the text to avoid overstating the role of the α1 subunit.

34) Line 433: “as a benzodiazepine-class compound, CL218872…” This compound is not a classical benzodiazepine. It would be better to refer to it as a benzodiazepine-site ligand, or a benzodiazepine-like/nonbenzodiazpine (z-drug) compound.

Response: We thank the reviewer for the precise pharmacological correction. We have revised the text to refer to CL218872 more accurately as a "compound acting through the benzodiazepine binding site" to avoid the inaccurate implication that it is a classical benzodiazepine in structure.

35) Line 435: “its concomitant use with opioids may exacerbate respiratory depression…” You should mention that opioids are relevant to this point because they are commonly prescribed after ICH.

Response: We thank the reviewer for this important suggestion for clarification. The point raised is highly relevant, as specifying the clinical context of opioid use following ICH better underscores the significance of this safety consideration. We have revised line 435 as suggested to explicitly state that opioids are "commonly prescribed for post-ICH analgesia."

36) Line 463: “The present study was approved by the Ethics Committee of Yunnan Beisitai Biotechnology Co.” I’m not sure if this is an error in translation or romanization, but I was unable to find any results for “Yunnan Beisitai Biotechnology” on any public databases.

Response: We thank the reviewer for their rigorous check on this detail. "Yunnan Beisitai Biotechnology Co., Ltd." is a legitimate contract research organization (CRO) based in Kunming, Yunnan, China, which holds the requisite accreditations for conducting animal experiments and operates an independent Animal Ethics Committee. Its SPF-level animal facility complies with Chinese regulatory guidelines. As its name is a romanization from Chinese, it may not be readily searchable in international databases. The

qualifications of the institution can be verified by accessing the following website: https://kjj.km.gov.cn/c/2022-12-16/4615697.shtml. We have verified the validity of its ethical review through the institution's certification documents. The figure below is certificate for Animals Experimental issuing by Kunming Science and Technology Bureau of Yunnan province on Dec.26th,2022, which is vail from Dec.,16th,2022 to Dec.,15th,2027.

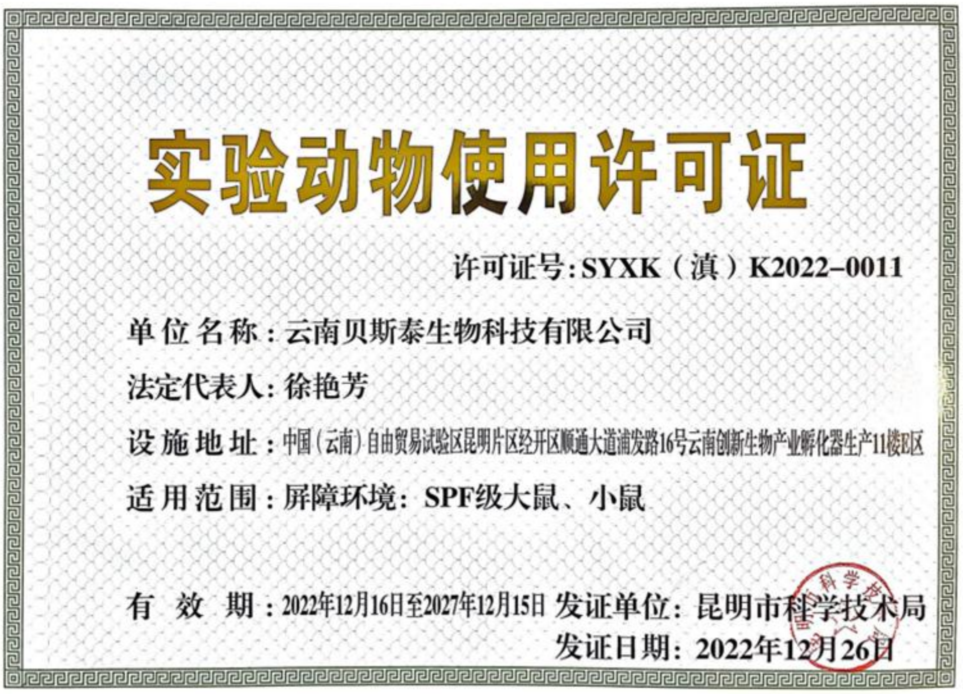

实验动物使用许可证

许可证号：SYXK（滇）K2022-0011

单位名称：云南贝斯泰生物科技有限公司

法定代表人：徐艳芳

设施地址：中国（云南）自由贸易试验区昆明片区经开区顺通大道浦发路16号云南创新生物产业孵化器生产11楼B区

适用范围：屏障环境：SPF级大鼠、小鼠

有效期：2022年12月16日至2027年12月15日 发证单位：昆明市科学技术局

发证日期：2022年12月26日

37) Line 468: “All data are in the manuscript and/or supporting information files" as your Data Availability Statement” – remove “as your data availability statement” from this.

Response: We thank the reviewer for pointing out the redundant phrasing. We have removed it.

38) Please double-check all citations, especially issue, volume and page numbers, as there are still many errors. Reference 7 for example lists the page as “13344.” Reference 10 only lists “16:1592277.” Reference 12 doesn’t have anything for these components, etc.

Response: We sincerely thank the reviewer for their meticulous examination of the references and for pointing out these issues. We sincerely apologize for these oversights. We have now systematically rechecked and corrected the volume, issue, and page numbers for all citations to ensure they are complete, accurate, and conform to the journal's formatting requirements. The specific errors in references 7, 10, 12,

among others, have been corrected. However, since volume numbers are not provided in some sources (such as Reference 33), we have not included volume numbers.

Reviewer #2: The authors have addressed most of my concerns, which were primarily with the lack of information provided about the two GABA-A ligands used in the study. The authors provided information about the pharmacology of the compounds, but the rationale for the choice of these ligands was not clear until the Discussion section, page 18. To increase clarity, I recommend moving the rationale to the Introduction section, where you first mention the compounds. These ideas can then be referred to in the Discussion (rather than repeated).

Response: We thank the reviewer for this constructive suggestion to improve the logical flow of our manuscript. We have followed the recommendation and moved the detailed rationale for selecting CL218872 and MRK-016 from the Discussion section to the Introduction, where these compounds are first introduced. In the revised Discussion, we now refer back to this foundational rationale rather than repeating it. We believe this modification significantly enhances the clarity and coherence of our manuscript's narrative.

Although it was stated that there was a revision to the Methods regarding how the behavioral scoring was done (blinding, etc.), I could not find these changes.

Response: We sincerely thank the reviewer for this careful observation, and we apologize that the methodological revisions regarding behavioral scoring were not immediately apparent. The detailed description of the blinding procedure has indeed been added to the "Neurological Deficit Score" subsection within the Methods section of the revised manuscript.

On page 7 and 8, the authors now describe the compound vehicle and provide a reference for dose. A more explicit statement about why these doses were chosen for each compound is still needed. Also, the description of dosing was confusing: "mice in the ICH+CL218872 group were administered a CL218872 and MRK-016 solution (5 mg/kg dissolved in DMSO) daily via gavage[25]." This reads as if CL218872 and MRK-016 were mixed together. Also, the reference does not appear to have MRK-016 in it. I assume that "5 mg/kg dissolved in DMSO" means that the ligands were dissolved in DMSO such that a 5 mg/kg dose was administered? Finally, it was stated that CL218872 was administered once per day. What about MRK-016?

Response: We sincerely thank the reviewer for raising these important points regarding pharmacological administration. We have thoroughly revised the Methods section accordingly. The description has been

modified to unambiguously clarify that CL218872 and MRK-016 were administered separately to their respective experimental groups and were never mixed together. We have also added an explicit rationale for the selected 5 mg/kg dose for both compounds. Finally, the text now clearly specifies that all drug administrations, including MRK-016, were performed once daily for 7 consecutive days.

Dr. Huan

Nov.,10th,2025

1. Please remove your figures/ from within your manuscript file, leaving only the individual TIFF/EPS image files. These will be automatically included in the reviewer's PDF

Response:

1. Figures were removed from the manuscript file.

I'm looking forward to receiving any news from you.

Sincerely yours,

Dr. Huan,

Nov.13th,2025
