# Supplementary material for: Effects of GABAAR modulators CL218872 and MRK-016 on neural repair and synaptic plasticity in mice with Intracerebral hemorrhage
Source: PLoS One. 2026 Mar 23;21(3):e0345025. doi: 10.1371/journal.pone.0345025 (PMC13008086; doi:10.1371/journal.pone.0345025)
Supplement: Attachment — Submitted filename: response letter20260104.pdf [file pone.0345025.s012.pdf]

Dear Paula Katrina A. Maderazo,

Thank you for your email, and we made an amendment point-by-point according to your comments.

Submission ID:PONE-D-25-22559R5

Title: Effects of GABAAR modulators CL218872 and MRK-016 on neural repair and synaptic plasticity in mice with Intracerebral hemorrhage

Editors’comments:

1. Please note that funding information should not appear in the Acknowledgments section/Funding section or Any other areas of your Manuscript. We will only publish funding information present in the Funding Statement section of the online submission form. Please remove any funding-related text from the manuscript.

Authors’Response:

1.Funding information has been removed from manuscript. Details please refer to line 609.

Kind regards，

Dr. Fei Huang

Jan.4th,2026

Dear Uwe Rudolph：

We are grateful for your meticulous review of this manuscript and your valuable suggestions. We have carefully considered each of your comments, and the relevant revisions are detailed in the revised manuscript titled ‘revised manuscript with track changes’. The key modifications and our responses to the reviewers' comments are outlined below:

1. Response to Reviewer 1

Major points:

The authors introduce a range of caveats regarding benzodiazepine administration in the discussion. While these are important considerations, especially the section

regarding stroke patients, this section is roughly 1 page long includes many clinical contexts not immediately related to ischemic injury, e.g. "Furthermore, this drug class increases the risk of cognitive decline and falls in elderly populations, may cause agitation and sleep disturbances in pediatric intensive care settings, and during pregnancy can lead to low birth weight, preterm delivery, and neonatal withdrawal syndrome[68]." As CL-218872 does indeed show neuroprotective properties after ICH in this model, the manuscript would be strengthened if more space were dedicated to contrasting CL-218872's mechanism of action, selectivity or dosing with the clinical use of classical (nonselective) benzodiazepines. For example, touching upon how generalized GABAergic inhibition may worsen clinical outcomes, but targeting inhibition to the alpha1-containing GABAARs or within the hippocampus may avoid these complications.

Author's response: We thank the reviewer for their comments. This study confirms that CL-218872 exhibits significant neuroprotective effects following cerebral haemorrhage. As requested by the reviewer, we have systematically compared this compound with classical non-selective benzodiazepines in the discussion section: CL-218872 maintains high specificity for α1-GABA_A receptors, exhibiting a manifold increase in therapeutic index at equivalent sedative doses. It circumvents common complications associated with traditional benzodiazepines, such as respiratory depression, hypotension, and delirium, thereby further consolidating the reliability of our findings.

Minor points:

1) Line 176: "α1-NAM" MRK-016 is an α5-NAM.

Author's response: Thank you for your comments. We have amended 'α1-NAM MRK-016' to 'α5-NAM MRK-016'.

2) Line 181-185: Please confirm the volumes between the collagenase (500uL) and sham mice (50uL saline) as I'm not sure if they were supposed to be the same.

Author's response: We thank the reviewer for their comments. To avoid confounding

factors such as volumetric injury and variations in intracranial pressure, the injection volumes for both the collagenase group and the sham surgery group should be consistent. The authors have amended the original typographical error, clarifying that 50 μL of collagenase corresponds to 50 μL of physiological saline.

3) Line 264: Please add RIPA (radioimmunoprecipitation assay) to the list of abbreviations.

Author's response: Thank you for your comments. The abbreviation RIPA and its full name have been added to the list of abbreviations.

4) Line 296: Change “Consistently with…” to “Consistent with”

Author's response: Thank you for your comments. We have amended ‘Consistently with’ to ‘Consistent with’.

5) Line 311: Change “the phosphorylation levels of p65…” “the levels of phosphorylated p65…”

Author's response: Thank you for your comments. We have amended ‘the phosphorylation levels of p65’ to ‘the levels of phosphorylated p65’.

6) Line 316: It’s stated that TNF-α expression is attenuated after ICH by CL-218872 and $p<0.05$ is cited at the end of the sentence. However, in figure 1E, there is no comparison made between the ICH and the ICH+CL-218872 groups. If significant, please amend the figure with this comparison and if not, please clarify this is a trend-level change.

Author's response: We appreciate the reviewer's comments. The $p < 0.05$ at the end of the sentence solely indicates a significant difference between the ICH group and the ICH+MRK-016 group; the ICH+CL-218872 group showed only a trend towards change compared to the ICH group. This statement has been amended in the main text.

7) Line 322, Fig 1C legend: Consider mentioning the concentrations for figure 1C are in pg/mL as well.

Author's response: We thank the reviewer for their comment. The data units have been clarified as pg/mL in the legend for Figure 1C.

8) Line 363: “p<0.01” is actually p<0.001 on the figure, please correct the text.

Author's response: Thank you for your comments. We have amended ‘p<0.01’ to ‘p<0.001’.

9) Line 365: “p<0.05” is actually p<0.001 on the figure, please correct the text.

Author's response: Thank you for your comments. We have amended ‘p<0.05’ to ‘p<0.001’.

10) Line 384: it’s stated the ICH group had “complete absence of synaptic vesicles,” however, 3 synaptic vesicles are labeled in the ICH image in figure 4. Please revise this.

Author's response: Thank you for your comments. The phrase ‘complete absence of synaptic vesicles’ has been amended to ‘marked reduction in synaptic vesicles’.

11) Line 393: Remove the extra “.” at the start of the line. “.Magnification”

Author's response: Thank you for your comments, reviewer. The full stop has been removed.

12) Line 400: “…showed significant upregulation of these markers (p<0.05),” clarify this is compared to the ICH group, “…upregulation of these markers compared to the ICH group (p<0.05).

Author's response: We thank the reviewer for their comments and have clarified the conclusion as follows: Compared with the ICH group, the marker was significantly upregulated.

13) Line 401: “reflecting exacerbated synaptic impairment,” as this is discussing markers of plasticity, it would be more accurate to say “reflecting/suggesting impaired synaptic plasticity”

Author's response: Thank you for your comments. The wording has been amended to: reflecting/suggesting impaired synaptic plasticity.

14) Line 426: “At the overall level, CL-218872…” change to “Overall, CL-218872…”

Author's response: Thank you for your comments. The phrase ‘At the overall level’ has been amended to ‘Overall’.

15) Line 428: “This finding not only directly validates the potential therapeutic value…” While the enhanced markers of plasticity, reduction in inflammation and improvement in synaptic structure signal the translational relevance of this approach, it is an overstatement to say it directly validates the therapeutic value and should be rephrased.

Author's response: We thank the reviewer for their comments. To rectify the appropriateness of this phrasing, we have amended ‘potential therapeutic value’ to ‘potential research value’.

16) Line 430: “…but also establishes a crucial functional correlation between improved neurological deficit scores and subsequent cellular/molecular mechanistic investigations.” It isn’t entirely clear what this sentence is arguing, as no correlational analysis was performed between NDS and marker expression or morphology, and the relevance of pro-inflammatory markers to ischemic injury have been previously-established (e.g. PMID: 27615422).

Author's response: We thank the reviewer for their comments. This argument indeed lacks direct empirical support. To ensure the scientific rigour and precision of the paper's claims, the point in question has been removed.

17) Line 439: “these findings demonstrate the CL-218872’s comprehensive hippocampal protection through maintaining cellular homeostasis…” please reword to “these findings demonstrate CL-218872’s comprehensive hippocampal protection is through maintaining cellular homeostasis…”

Author's response: Thank you for your comments. The revisions have been made as requested.

18) Line 444-447: This first sentence is redundant with the following sentence on lines 447-450, restating many of the same findings, and can be removed.

Author's response: We thank the reviewer for their comments. There were indeed multiple instances of similar conclusions in this section, and the authors have now made the necessary deletions.

19) Line 449: “…exerting anti-inflammatory effects” change to “…exerts anti-inflammatory effects.”

Author's response: Thank you for your comments. We have amended the phrase ‘exerting anti-inflammatory effects’ to ‘exerts anti-inflammatory effects’.

20) Line 463: Change “…significant modulatory effect” to “significant effect.”

Author's response: Thank you for your comments. The phrase ‘significant modulatory effect’ has been amended to ‘significant effect’.

21) Line 446: “showed an upregulating trend” change to “was (significantly) upregulated.”

Author's response: Thank you for your comments. We have amended the phrase ‘showed an upregulating trend’ to ‘was (significantly) upregulated’.

22) Line 487: “…the overall regulation of neural network E/I balance by CL-218872…” clarify this is toward enhanced inhibition, e.g. “the shift toward enhanced inhibition produced by CL-218872…”

Author's response: We thank the reviewer for their comments. The regulatory role of CL218872 on neural networks has been explicitly clarified as an inhibitory effect.

23) Line 489: Although the citation (55) provided discusses astrocytes, it primarily discusses them in the context of mechanisms related to fatty acids and does not appear to support the assertion that shifts in the E/I balance can facilitate astrocytic transition from A1 to A2 states. Please provide a new citation for this line.
Author's response: Thank you for your comments. The latest references supporting this argument have been added.

24) Line 490: "Regarding synaptic structure and functional restoration," this study did not directly examine the function of hippocampal neurons and thus the reference to functional restoration should be removed.
Author's response: Thank you for your comments. The phrase 'functional recovery' has been removed, retaining only 'structural recovery'.

25) Line 491: "…clear promotive effect…" change to "protective/neuroplastic effect"
Author's response: Thank you for your comments. We have amended 'clear promotive effect' to 'protective/neuroplastic effect'.

26) Line 521: "in an intracerebral hemorrhage (ICH) model." As the acronym ICH has already been used in the discussion, you can remove "intracerebral hemorrhage" here.
Author's response: Thank you for your comments. The full name of ICH has been removed, with only the abbreviation retained.

27) Line 565: "alongside their therapeutic benefits." Please touch upon the specific therapeutic benefits of benzodiazepines in the context of ICH.
Author's response: We thank the reviewer for their comments. The therapeutic effects of benzodiazepines have been specified as follows: they can rapidly control

seizures and intracranial pressure during the acute phase of cerebral haemorrhage.

28) Discussion: In the discussion, the word “synaptophysin” is capitalized throughout. Please change to lower-case.

Author's response: Thank you for your comments. The entire manuscript has been revised accordingly.

29) Discussion: while the authors discuss bidirectional modulation of GABAergic transmission via positive allosteric modulation at alpha1-containing and negative modulation at alpha5-containing receptors, as mentioned in the manuscript, these receptor subtypes contain different populations of GABAARs, and also generally mediate phasic vs. tonic inhibition. It would be worthwhile to touch upon these mechanistic differences in the discussion, e.g. comparing MRK-016 to an alpha5-selective GABA-PAM to bidirectionally modulate the same populations of GABAARs. This would also be an interesting follow-up in a future work.

Author's response: We appreciate the reviewer's comments. α1, α5 and other receptor subtypes indeed belong to distinct GABAAR subfamilies, exhibiting marked differences in their underlying inhibitory mechanisms. Consequently, the discussion section has clarified that α1 and α5 subtypes, among others, belong to separate GABAAR subfamilies, mediating transient phase inhibition and sustained tonic inhibition respectively. Based on this, we propose their bidirectional regulation as a novel strategy for future research.

30) Abbreviations: Remove GABAbR as it does not appear in the manuscript.

Author's response: Thank you for your comments. The reference has been removed from the abbreviations table.

2. Response to Reviewer 2

Reviewer 2 has no further comments on this manuscript.

The foregoing constitutes the author's complete explanation regarding this revision. Once again, we extend our sincere gratitude for your meticulous review and invaluable guidance.

Kind regards，

Dr. Fei Huan
Corresponding author
E-Mail：mzkhuangfei123@163.com
