# Supplementary material for: Effects of GABAAR modulators CL218872 and MRK-016 on neural repair and synaptic plasticity in mice with Intracerebral hemorrhage
Source: PLoS One. 2026 Mar 23;21(3):e0345025. doi: 10.1371/journal.pone.0345025 (PMC13008086; doi:10.1371/journal.pone.0345025)
Supplement: Attachment — Submitted filename: Response to Reviewers20260123.pdf [file pone.0345025.s013.pdf]

Dear Uwe Rudolph,

We extend our heartfelt gratitude for your meticulous review of the manuscript and invaluable guidance. Each of your comments has been thoroughly examined and considered, with responses and revisions implemented point by point based on relevant literature and experimental data. All modifications are clearly annotated in the ‘Revised Manuscript with Track Changes’ file, enclosed for your further review. Should any shortcomings remain, we would be most grateful for your continued guidance.

Comments 1： Line 384: “a marked absence of synaptic vesicles” – as requested before, please reword to “a marked reduction” or something similar, as you label vesicles in the ICH group in figure 4, so they are not truly “absent.”

Response： We are grateful for the reviewer's comments and fully concur with this observation. Consequently, we have amended ‘a marked absence’ to ‘a marked reduction’. Please refer to line 384 of the revised manuscript for the specific change.

Comments 2： Line 428: “this finding directly confirms that…” – the wording on this should be softened, e.g. “this finding supports…”

Response： We are grateful for the reviewer's comments and fully concur with this observation. Consequently, we have revised the phrasing from ‘this finding directly confirms that’ to ‘this finding supports’. Please refer to line 430 of the revised manuscript for the specific amendment.

Comments 3： Line 437: capitalize the T in “These”

Response： We thank the reviewer for their comment and have amended the lowercase “t” to uppercase “T”. Please refer to line 439 of the revised manuscript for the specific change.

Comments 4： Line 557: “MRK-016 (an α5-non-agonist) and α5-agonists exhibit mirror-image bidirectional regulation of the same α5 subunit” – it is more accurate to

refer to MRK-016 as an “inverse agonist,” not a non-agonist. Furthermore, I would remove the phrase “mirror-image” as the binding affinity and efficacy between MRK-016 and other α5-selective GABA-PAMs vary widely. Additionally, these compounds don’t regulate the subunit itself, rather, they regulate signaling/transmission through GABAARs containing the α5 subunit.

Response： We are grateful for the reviewer's comments and fully concur with this observation. This revision indeed more rigorously reflects the differences in affinity and efficacy among various α5-selective ligands. Furthermore, specifying the target as ‘GABAA receptor signalling involving the α5 subunit’ rather than the more general ‘regulation of the subunit itself’ better aligns with the pharmacological mechanism. Accordingly, we have made the following amendments:

1) Replace ‘non-agonist’ with ‘inverse agonist’ to accurately describe MRK-016's pharmacological properties;

2) Remove the concept of ‘mirroring’ to avoid ambiguity arising from differences in binding affinity and efficacy between compounds.

3) Replace ‘regulation of the α5 subunit itself’ with the more precise ‘regulation of signalling/transduction via GABAA receptors containing the α5 subunit’ to accurately reflect the mechanism of action.

Please refer to line 564 of the revised manuscript for specific details.

Comments 5： Line 558: “This principle may be leveraged to design MRK-016 for comparative studies with α5-selective GABA-agonists, examining their bidirectional regulatory effects on the same GABAAR subunit[65]” – The way this is phrased is unclear (suggests manipulating the MRK-016 molecule?). I’d suggest rewriting this similar to “The GABA-NAM MRK-016 can be used in comparative studies with α5-selective GABA-PAMs to examine the effects of bidirectional manipulation of α5-containing GABAAR function on… etc”

Response： We are grateful for the reviewer's comments and fully concur with this observation. Consequently, we have rewritten the original sentence as suggested to avoid any potential ambiguity regarding the ‘design of MRK-016’. The revised

wording is as follows:

Accordingly, GABA-NAM MRK-016 may be employed in combination with α5-selective GABA-PAMs for comparative studies to investigate the bidirectional regulatory characteristics exhibited by GABAA receptors containing the α5 subunit in functional terms. Please refer to line 566 of the revised manuscript for details.

Comments 6: Paragraph starting at line 561: Consider consolidating the descriptions of side-effects of benzodiazepines, as this section is still relatively long (about an entire page). For example, this sentence can be removed as it is mostly redundant with the previous: "Long-term administration frequently leads to psychological and physiological dependence, with withdrawal symptoms mirroring those of alcohol withdrawal—including anxiety, insomnia, tremors, and seizures—that may become life-threatening in severe cases." You can then combine this with the previous sentence: "…yet they also carry significant risks of dependence, adverse cognitive effects, and withdrawal reactions like anxiety, insomnia, tremors and seizures."

Response: We are grateful for the reviewer's comments and fully concur with this observation. Accordingly, we have consolidated the first three sentences of the paragraph as per the reviewer's suggestion to sharpen the focus. Furthermore, we have streamlined and integrated subsequent literature references, such as: 'Moreover, this drug class elevates the risk of cognitive decline and falls in elderly populations...' to address redundant and verbose descriptions within the paragraph. In summary, the revised section now falls within one page. Please refer to lines 573–623 of the revised manuscript for the specific changes.

Comments 7: Line 575: "Clinical study demonstrated…" -- change to "A clinical study demonstrated…"

Response: We thank the reviewers for their comments. We have amended the phrasing from 'Clinical study demonstrated' to 'A clinical study demonstrated' in accordance with the reviewers' suggestions. Please refer to line 583 of the revised manuscript for the specific change.

Comments 8: Line 579: “These findings do not support any putative neuroprotective effects of GABAA receptor agonists” – change to “nonselective GABAA receptor agonists” to provide distinction with the CL-218872 compound and classical benzodiazepines in the study.

Response: We are grateful for the reviewer's comments. We have made the necessary amendments in accordance with the reviewer's suggestions. Please refer to line 586 of the revised manuscript for the specific changes.

Comments 9 : Throughout the manuscript: Please standardize the naming of CL-218872 throughout the paper, as it is referred to as both “CL218872” and “CL-218872” in different sections.

Response: We thank the reviewers for their comments. We have now standardised the reference throughout the text as ‘CL218872’.

Kind regards,

Dr. Fei Huang

Corresponding Author

E-Mail: mzkhuangfei123@163.com
